# From co-creation to practice: a collaborative program strengthening palliative care capacity in primary health care in Ghana

**DOI:** 10.3389/fmed.2026.1848734

**Published:** 2026-07-29

**Authors:** Mawuli Kotope Gyakobo, Abigail Kusi Amponsah, Michael Owusu-Ansah, Anita Eseenam Agbeko, Perditer Okyere, Arti Singh, Amy Nolen, Adam Goldman, Rachel Wortzman, Sandy Shamon, Jamie Meuser, Paolo Mazzotta, Katherine Whitehead, Salman Bahammam, Princess Ruhama Acheampong, Eric Adjei Boadu, Kofi Akohene Mensah, Ellis Owusu-Dabo, Jennifer Wilson, Katherine Rouleau, Kirsten Wentlandt

**Affiliations:** 1 Department of Internal Medicine and Therapeutics, School of Medical Sciences, College of Health and Allied Sciences, University of Cape Coast, Cape Coast, Ghana; 2 Department of Public Health Nursing, School of Nursing and Midwifery, College of Health Sciences, Kwame Nkrumah University of Science and Technology (KNUST), Kumasi, Ghana; 3 Department of Community Health, School of Medical Sciences, College of Health Sciences, KNUST, Kumasi, Ghana; 4 Department of Surgery, School of Medical Sciences, College of Health Sciences, KNUST, Kumasi, Ghana; 5 Renal Unit, Department of Medicine, School of Medical Sciences, College of Health Sciences, KNUST, Kumasi, Ghana; 6 Department of Epidemiology and Biostatistics, School of Public Health, College of Health Sciences, KNUST, Kumasi, Ghana; 7 Division of Palliative Care, Department of Family and Community Medicine, University of Toronto, Toronto, ON, Canada; 8 Division of Palliative Medicine, Sunnybrook Health Sciences Centre, Toronto, ON, Canada; 9 Division of Palliative Care, Temmy Latner Centre for Palliative Care, Sinai Health System, Toronto, ON, Canada; 10 Palliative Medicine Unit, Department of Internal Medicine, King Saud University, Riyadh, Saudi Arabia; 11 Department of Health Promotion and Disability Studies, School of Public Health, College of Health Sciences, KNUST, Kumasi, Ghana; 12 Department of Population, Family and Reproductive Health, School of Public Health, College of Health Sciences, KNUST, Kumasi, Ghana; 13 Department of Health services Planning and Management, School of Public Health, College of Health Sciences, KNUST, Kumasi, Ghana; 14 Department of Global and International Health, School of Public Health, College of Health Sciences, KNUST, Kumasi, Ghana; 15 Department of Family and Community Medicine, University of Toronto, Toronto, ON, Canada; 16 St. Michael's Hospital, Unity Health Toronto, Toronto, ON, Canada; 17 WHO Collaborating Center on Family Medicine and Primary Care, Toronto, ON, Canada; 18 Division of Palliative Care, Department of Supportive Care, University Health Network, Toronto, ON, Canada

**Keywords:** capacity building, co-creation, global health, interprofessional education, palliative care, primary health care, program development, sustainable development goals

## Abstract

Access to palliative care in Ghana remains limited, with services concentrated in urban centers and few healthcare professionals trained in this area. Within a primary health care (PHC) framework, strengthening palliative care capacity at the frontline is essential to achieving equitable access and achieving Universal Health Coverage (UHC). To address this gap, Kwame Nkrumah University of Science and Technology and the University of Toronto co-created a short course in palliative care for primary care providers, grounded in adult learning principles and experiential pedagogy. Between 2023 and 2025, three cohorts completed two in -person palliative care educational modules, each spanning 3–4 days, using a curriculum that combined didactic teaching, case-based learning, role play, reflective exercises, and site visits. Formative program evaluation using a mixed methods approach included daily surveys, three end-of-course questionnaires, and six focus group discussions. Quantitative data were analyzed descriptively, while qualitative feedback underwent thematic analysis. 65 participants were enrolled, predominantly nurses (*n* = 45, 69.2%), followed by physician assistants (*n* = 9, 13.8%), midwives (*n* = 7, 10.8%), and smaller numbers of other allied health professionals (*n* = 4, 6.2%). The majority were female (*n* = 35, 53.8%), and most were early to midcareer clinicians (*n* = 51, 78.5% under age 40). Daily evaluations showed high satisfaction, with over 95% reporting lessons were clear and relevant, and 98% indicating they would recommend the course to others. Focus group feedback highlighted improved confidence in serious illness communication, patient advocacy, and holistic care, alongside requests for longer training duration, more clinical exposure, and ongoing mentorship. Learners expressed readiness to integrate palliative care into their practice, with many reporting concrete plans to establish or expand services in their facilities. Findings are, unfortunately, limited by reliance on short-term, self-reported outcomes, without objective assessment of behavior change or patient-level impact. This formative program evaluation demonstrates that co-created training can strengthen palliative care capacity in resource constrained settings. By prioritizing equity in recruitment and embedding experiential learning, the program fostered readiness among frontline providers to integrate palliative care within primary care settings, contributing to more equitable person-centered care, and advancing UHC.

## Introduction

Primary health care (PHC) is a whole-of-society approach that forms the foundation of equitable, effective health systems and a key strategy for achieving Universal Health Coverage (UHC) and the health-related Sustainable Development Goals (SDGs) (1–5). Within this context, the World Health Organization (WHO) recognizes that palliative care is an essential component of comprehensive, person-centered care across the life course. Palliative care strengthens health systems' capacity to reduce suffering, improve quality of life, and advance equity, and is therefore critical to PHC, and to achieving UHC and broader development goals.

Palliative care focuses on improving the quality of life for patients with life-limiting illnesses and their caregivers/families by addressing their physical, emotional, spiritual, and social needs (6). The demand for palliative care has increased due to the rising prevalence of non-communicable diseases (NCDs), increased life expectancy, a growing elderly population susceptible to chronic illness, emerging awareness of palliative care needs, and improved survival among children and adults with complex medical conditions (4).

Despite the growing need for palliative care services, a significant global disparity persists in access, especially in developing economies. Globally, an estimated 56.8 million people require palliative care annually, yet only about 14% of these individuals receive direct care (6). Although access to palliative care has been deemed a human right, most people living in low- and middle-income countries lack sufficient access due to human and material resource constraints (5, 6). The situation is dire in sub-Saharan Africa amidst its rising prevalence of NCDs and access to palliative care still in its infancy (7–9).

In Ghana, palliative care services remain limited, and available services are inadequate to meet the needs of the population (10). Currently, comprehensive palliative care is provided by only six out of 2,773 health facilities in Ghana, namely: Komfo Anokye Teaching Hospital (KATH), Tetteh Quarshie Memorial Hospital (TQMH), Korle Bu Teaching Hospital (KBTH), Greater Accra Regional Hospital (Ridge Hospital), Matthew 25 Hospice-Koforidua, and Peace and Love Hospital-Kumasi (11, 12). Among these, KBTH and KATH are the only facilities with well-established palliative care programs; however, these facilities lack dedicated in-patient care facilities (13, 14). TQMH is the only hospital that operates a comprehensive and dedicated in-patient palliative care facility, in addition to out-patient practice. There is an inequitable distribution of palliative care services, as all four health facilities are concentrated within urban areas in the southern part of the country. The limited provision of palliative care in Ghana can be partly attributed to the insufficient number of healthcare professionals trained in this area (11, 15).

Educating health care professionals (HCPs) to provide high-quality palliative care is a recognized and crucial part of integration efforts, given the disparities in palliative care availability worldwide (16). Although recent efforts have seen palliative care incorporated into the curricula for nursing and medical students (13), other allied health professionals are yet to formally incorporate it into their educational journey. This lack of multidisciplinary training further limits the healthcare system's ability to provide holistic care for patients. In addition, studies indicate that many HCPs in Ghana interpret palliative care as simply end-of-life care, rather than a holistic approach to managing chronic or life-threatening illnesses and improving quality of life, often leading to late referrals for palliative care services (15, 17). These factors suggest the need for transformative education in palliative care to correct misconceptions and improve outcomes for all stakeholders. Recent efforts to address palliative care gaps in Ghana and sub-Saharan Africa have increasingly emphasized education and training of healthcare providers, including the integration of palliative care into undergraduate medical and nursing curricula and the development of short-course training initiatives (13, 15, 17). However, existing programs have largely focused on didactic approaches, with limited opportunities for experiential learning, mentorship, and practice-based skill development (18). In addition, many initiatives remain discipline-specific, with less emphasis on team-based or system-level capacity building, and few studies report on sustained practice change or patient-level outcomes (19, 20). These gaps highlight the need for more comprehensive, contextually grounded training models that combine clinical skill development, reflective practice, and implementation-oriented learning to support the integration of palliative care into routine care delivery.

Given the substantial gap in palliative care services and the vital role HCPs play in delivering these services, there is an urgent need for educational programs targeted at a broader range of HCPs. By equipping HCPs with the skills and knowledge to offer palliative care, such a program can help address the inadequacies in palliative care service provision in Ghana. Moreover, it will focus on improving the quality of life for patients with serious illness along with their respective caregivers and support the broader goal of achieving UHC. In response to these gaps, a North–South academic partnership between Kwame Nkrumah University of Science and Technology (KNUST) and the University of Toronto (UoT) was established to combine complementary expertise in palliative care education with local contextual knowledge, enabling the co-creation of a program tailored to Ghana's health system needs.

### Kwame Nkrumah University of Science and Technology and University of Toronto Collaboration

The Africa Higher Education Health Collaborative is a multi-year partnership between eight African higher education institutions, the UoT, and the Mastercard Foundation. Each partner contributes unique strengths and expertise to drive sustainable growth and transformation of Africa's health sector while improving regional health outcomes. As part of this initiative, KNUST in Ghana is collaborating with the Department of Family and Community Medicine (DFCM) at UofT to co-create and co-deliver five continuing education programs designed to enhance the capacity of primary care providers in Ghana, address evolving health system needs and align with national PHC priorities. One of these programs focuses on the delivery of palliative care.

In 2023, faculty from KNUST and UofT partnered to design and deliver an innovative palliative care curriculum, delivered in two modules, each spanning 3–4 days of learning. In this article, we report on the development process of this program while examining the experiences of learners. Through reflections on co-creation, learning outcomes, and the application of acquired knowledge, this study seeks to provide insights that will inform and guide future educational initiatives in similar contexts. To address gaps in primary care palliative care capacity, this initiative focused on developing an educational intervention tailored to frontline providers within the Ghanaian health system. This study aims to describe the development, delivery, and evaluation of a co-created palliative care training program for primary care providers, and to examine learners' experiences and perceived readiness to apply palliative care in practice.

### Pedagogical framework and learning environment

As part of this Curriculum, Instruction, and Pedagogy (CIP) approach, evaluation strategies were embedded within the educational design to support iterative learning, reflective practice, and continuous course refinement. To outline the program's goals, processes, activities, outcomes, and outputs, we employed a mixed-method formative evaluation process using surveys and focus groups with learner participants. These embedded evaluation processes functioned as both assessment tools and pedagogical mechanisms, reinforcing learning through reflection and continuous feedback. The evaluation approach recognized the significance of process-based evaluation and attempted to capture both intended and emerging processes (21). The focus of this evaluation was on the pedagogy of the short course experience and the perceived learning from the viewpoints of participants.

### Course development and co-creation

**Figure 1:**
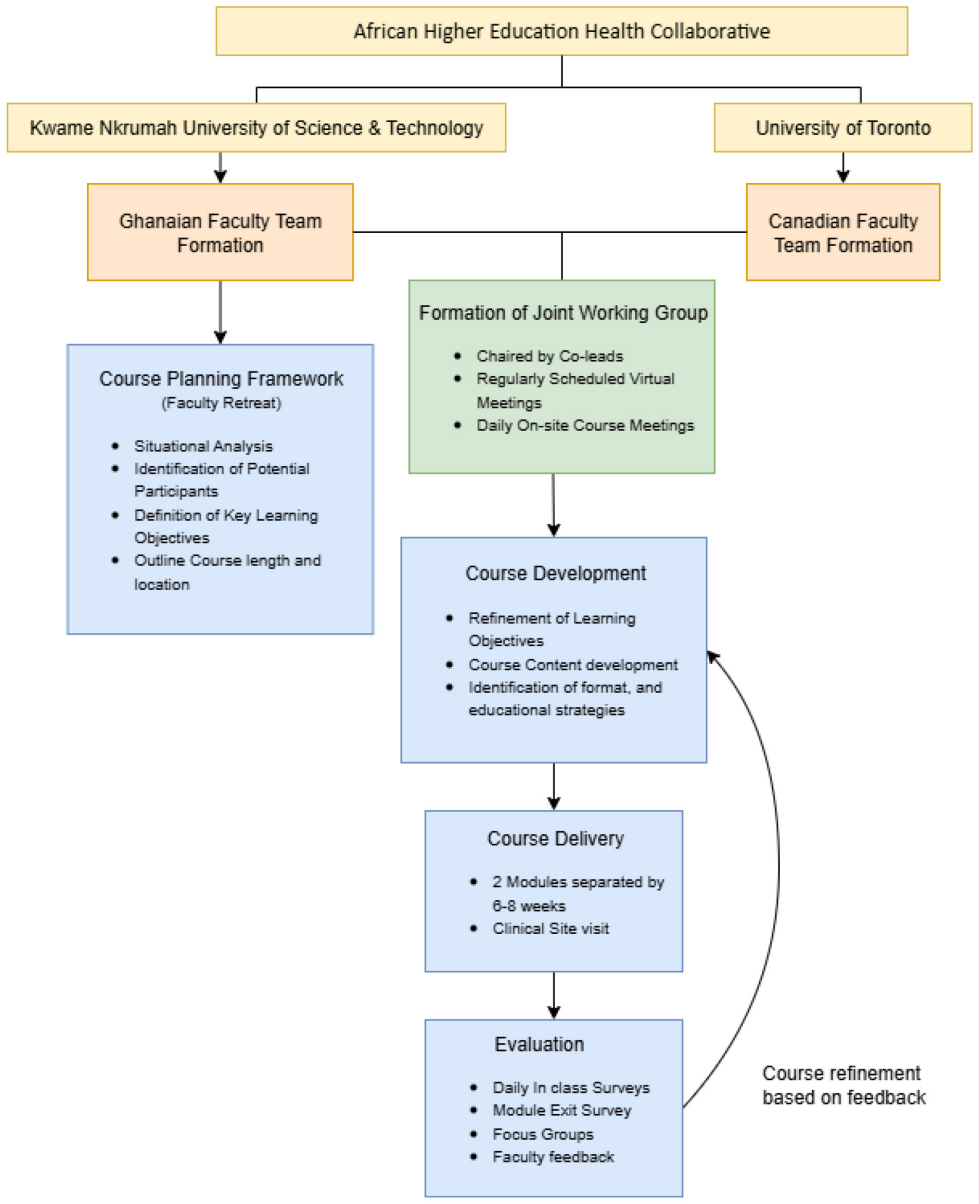
Co-creation process and course content.

Faculty members with expertise in palliative care, focused practice experience, or content expertise in specific topics were identified from KNUST and UofT (Figure 1, Table 1). Ghanaian faculty had appointments in Palliative Care, Internal Medicine, Family Medicine, Nursing and Midwifery, Surgery and Public Health. Canadian faculty had affiliations with Family Medicine and Palliative Care. Collectively, the joint faculty represented a comprehensive spectrum of palliative care practice in various care settings including community care, hospice, acute care and long-term care. Most faculty members had active academic practices involving research and teaching. Once faculty members were identified, a working group was established, and virtual meetings and electronic correspondences were utilized to initiate the co-design process and ultimately develop the content of the course as well as programming and final delivery. Content planning by involved faculty started approximately 6 months before the first module of the initial course which was delivered in September 2023. Regularly scheduled virtual meetings took place amongst faculty from each university as well as intermittent joint meetings involving both groups. The frequency of the co-design meetings increased from monthly in the early stages to biweekly then weekly toward the date of course delivery. After the delivery of each module, the faculty debriefed in person in Ghana and resumed virtual meetings later to discuss the evaluation process and future improvements.

**Table 1:** Combined topics covered in module I and module II.

Clinical symptoms management	Comprehensive person-centered assessment	Ethics, law and decision making	Epidemiology and health systems	Special topics
•Pain •Dyspnea •Nausea & Vomiting •Delirium •Depression & anxiety •Sleep & fatigue •Nutrition & anorexia •Skin care	•Whole person assessment •Principles of palliative care •Communication •Advance care planning •Serious illness conversations •Spiritual assessment	•Introduction to ethics •Medical futility •Withdrawing & withholding treatment •Physician-assisted suicide •Substitute decision makers	•Research methods •Data management •models of care •Development of palliative care program	•Pediatric palliative care •Palliative care emergencies •Last hours of life

The modules of the short course in palliative care were delivered in two parts; Module I and Module II. The didactic content of each module was delivered over 3 days (Figure 1). In 2024, based on participant and co-creator feedback, an additional day was added to Module 1, to include a site visit to a local hospital.

### Learning theory, pedagogy, and course content

The course was grounded in adult learning principles, emphasizing experiential, interactive, and practice-based approaches to support the development of clinical competence and reflective capacity in palliative care. A blended pedagogical model was used, combining didactic teaching, interactive group sessions, case-based learning, role-play, and reflective exercises. Learners also participated in clinical observation and patient/caregiver interactions during site visits, enabling the integration of theoretical knowledge with real-world practice. Throughout the course, iterative feedback, group problem-solving, and practical exercises, including hands-on activities in research methods, data management, and program development, supported adaptive learning and skill reinforcement.

The program design was also informed by principles aligned with decolonizing approaches to global health education, which emphasize co-creation, local leadership, and reciprocal knowledge exchange. Drawing on pedagogical traditions such as Freire's concept of dialogical learning (22) and critiques of extractive research paradigms (23, 24), the partnership prioritized collaborative curriculum development with Ghanaian faculty as equal contributors, ensuring that content, delivery, and learning priorities were grounded in local context and health system needs. This approach aimed to move beyond unidirectional knowledge transfer toward a model of mutual learning and shared ownership (25, 26).

The curriculum covered the full spectrum of palliative care knowledge and skills. Core clinical topics included symptom management (pain, dyspnea, nausea and vomiting, delirium, depression and anxiety, sleep and fatigue, nutrition and anorexia, and skin care) and comprehensive, whole-person assessment. Learners were introduced to foundational concepts in the principles of palliative care, alongside modules on communication, advance care planning, serious illness conversations, and spiritual assessment. Ethical and legal frameworks were addressed through sessions on medical futility, withdrawing and withholding treatment, physician-assisted dying, and the role of substitute decision makers. Supporting content included epidemiology and health systems, models of care, program development, research methods, data management, palliative care emergencies, and additional modules on the last hours of life. Together, these components created a comprehensive, competency-based learning experience designed to build clinical expertise, strengthen communication skills, and support the integration of palliative care principles into learners' diverse practice settings.

Minimal changes to the content were made from 2023 to 2024. Most changes involved re-ordering of topics and content revision related to feedback from participants. In 2024, a site visit was added to Module 1 where learners and teaching faculty conducted a site visit of KATH in Kumasi. During the site visit, learners observed and interacted with persons living with serious illness and practiced their communication skills by working with patients in various clinics (palliative care, oncology, nephrology and dialysis clinics, breast clinic, and internal medicine/ family medicine wards). Under faculty observation, the learners conducted goals of care and advance care planning conversations and reviewed pain and symptom management. To solidify the learning objectives, learners debriefed with faculty in real time and later in the classroom.

### Learner selection

The program was widely advertised through the university's communication channels and disseminated to hospitals and clinical providers across rural regions to ensure broad access and awareness. Interested applicants were required to complete a written application and submit a brief personal reflection describing the importance of the course for their professional development and the communities they serve. To promote equity and strengthen palliative care capacity where it is most needed, a structured point-based selection process was used. To support the programs' commitment to engaging early-career providers and advancing gender equity within the palliative care workforce. Applications were reviewed by a team of Ghanaian faculty using a structured, matrix-based scoring system, which assigned points based on predefined criteria including prioritizing practice in rural or underserved settings, early-career status, and gender equity. Selection decisions were made by consensus, and the same criteria were applied consistently across all cohorts. This approach ensured that course enrollment aligned with national priorities for health system strengthening, program priorities, and maximized the potential impact of the training across diverse care environments.

### Course evaluation

An evaluation survey was distributed to all participants, and they were invited to take part in focus group sessions at the start or end of the second module. The surveys were anonymous and included questions on basic demographics and prior experiences. Participants were asked to share their reflections and insights on the effectiveness of the learning activities, as well as provide suggestions for improving future iterations. The surveys were conducted using Qualtrics^®^, a software platform designed for data management and analysis (27). Mentimeter^®^ was used to help teach and collect daily feedback from participants during the course. (28)

The focus group guides were revised with feedback from KNUST and UofT stakeholders, as well as input from the KNUST data collection team. Focus groups were semi-structured, allowing for flexibility in responses and the emergence of new themes. Responses were recorded, anonymized, and transcribed by the data collection teams before analysis. Due to the focus group format and anonymization procedures, participant-level demographic identifiers (e.g., profession, cohort, or gender) were not linked to individual transcripts, and quotations are therefore presented without attribution to specific participant characteristics.

Qualitative data were analyzed using reflexive thematic analysis following the approach described by Braun and Clarke (29). Analysis focused on learners' feedback on course structure, duration, and content, as well as their reflections on the application of learning to clinical practice. Initial coding was conducted independently, with codes iteratively reviewed by 2 team members and refined to develop a shared coding framework. Discrepancies in coding and theme interpretation were discussed and resolved through consensus. Given the semi-structured design of the focus groups and the targeted nature of the discussion guide, the analysis was primarily descriptive and focused on summarizing key areas of participant experience and application of learning, rather than exploring a wide range of divergent viewpoints.

Survey and focus group interview data were analyzed using descriptive statistics and thematic analysis to summarize the open-ended responses. Participation in program evaluation activities, including surveys and focus groups, was voluntary. Informed verbal consent was obtained from all participants prior to data collection. Participants were informed of the purpose of the evaluation, the voluntary nature of participation, and the confidentiality of their responses. This study was approved by the KNUST Committee on Human Research, Publication and Ethics (ID: CHRPE/AP/1225/24).

## Results

### Learner demographics

The demographic characteristics of the 65 participants is provided in Table 2. Participants were predominantly early to midcareer, with 30.8% aged 20–29 years, 47.7% aged 30–39 years, and 21.5% aged 40 years or older. The majority were female (53.8%). Professional backgrounds reflected mainly representation from frontline clinical providers: nurses comprised 69.2% of participants, followed by midwives (10.8%), physician assistants (13.8%), and smaller proportions of health assistant clinical staff (1.5%), health service administrators (1.5%), nutrition officers (1.5%), and medical laboratory scientists (1.5%). Physicians and Pharmacists were not represented in the trained participants across all 3 cohorts and identified a recruitment goal for future cohorts.

**Table 2:** Participant demographics (*n* = 65).

Demographic	Number	Percentage
Age		
20–29 years	20	30.8%
30–39 years	31	47.7%
40 + years	14	21.5%
Gender		
Female	35	53.8%
Profession		
Nurse	45	69.2%
Midwife	7	10.8%
Physician assistant	9	13.8%
Health assistant clinical	1	1.5%
Health service administrator	1	1.5%
Nutrition officer	1	1.5%
Medical lab. scientist	1	1.5%

**Figure 2:**
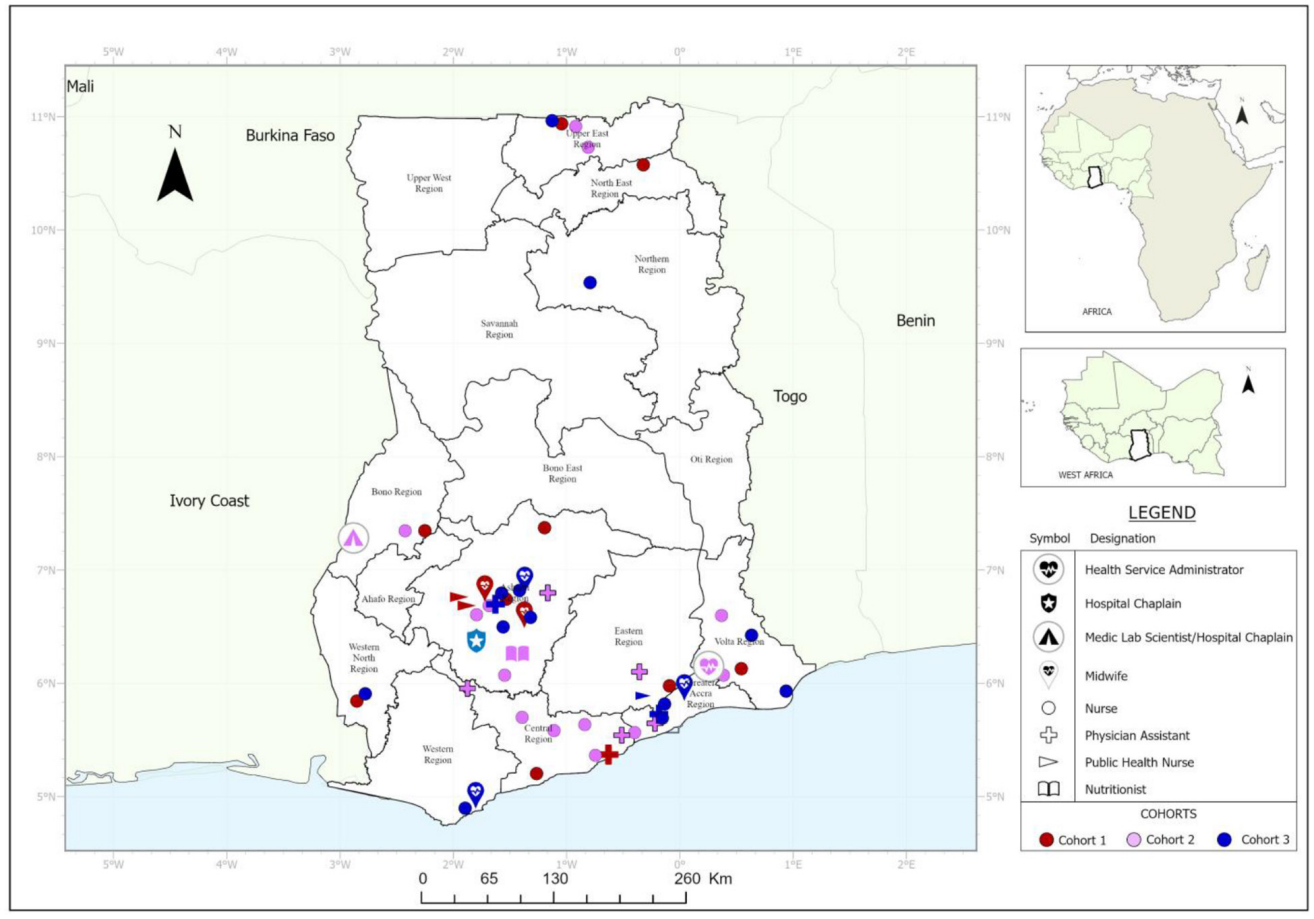
Map of participants (*n* = 65).

Figure 2 shows the distribution of course participants by profession across Ghana (*n* = 65). The Ashanti region where the host institution is located registered the highest number of participants and greatest professional mix. The Greater Accra (5), Central (8) and Volta (6) regions each registered about half as many participants compared to the Ashanti region. Participants from the latter 3 regions were predominantly nurses. Very few participants came from the Western (2), Western North (2), Bono (3), Northern (1), North East (1) and Upper East (4) regions. Participants from these regions were largely nurses as well. No participant came from the Upper West, Savannah, Oti, Bono East and Ahafo regions.

### Quantitative evaluation: in class survey data

**Figure 3:**
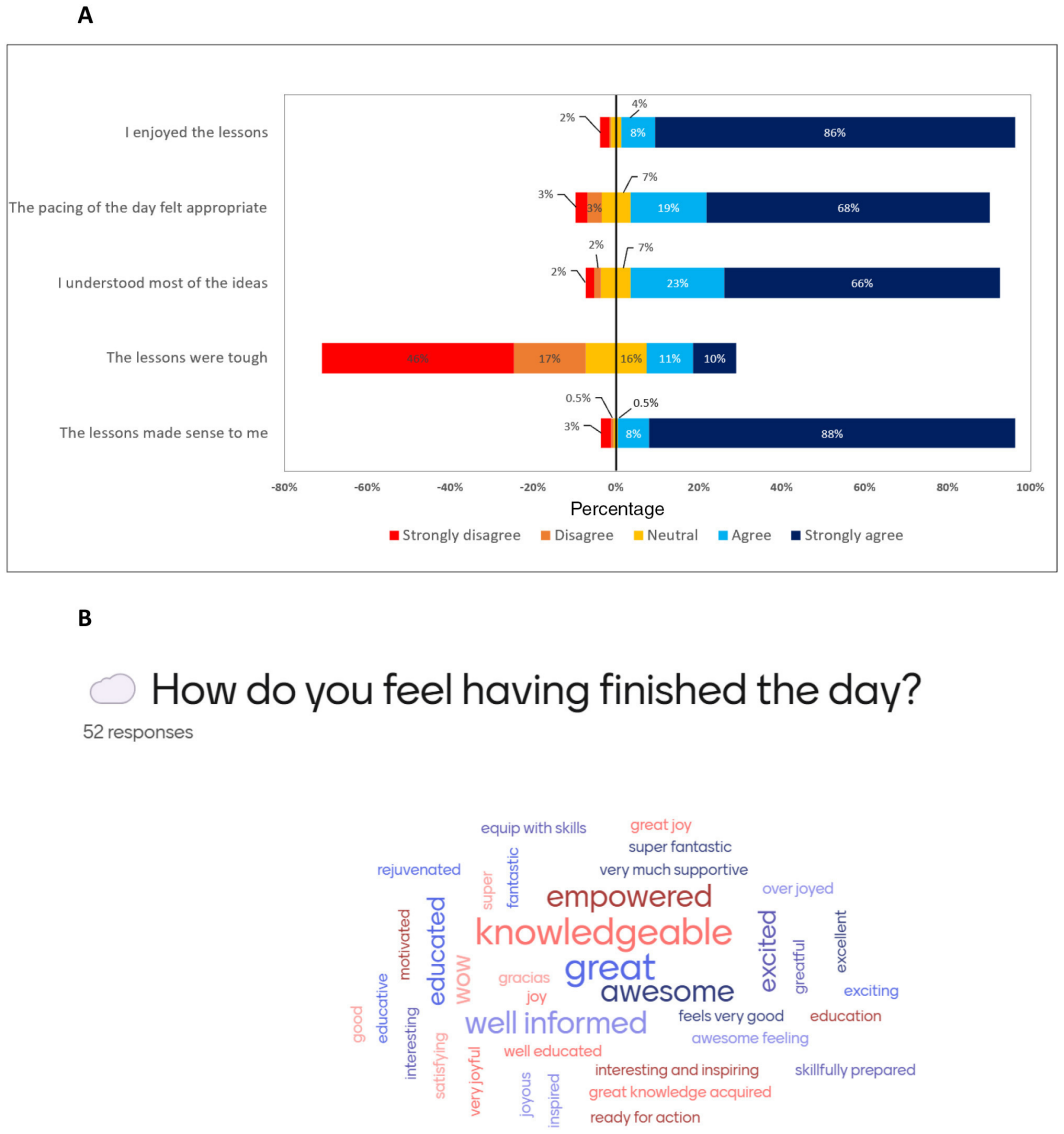
In class evaluations, 2023–2025 (*n* = 306).

In total, 306 daily Mentimeter responses were collected across sessions and aggregated for descriptive analysis of learner experience. Daily end-of-class evaluations from 2023–2025 demonstrated consistently showed strong learner satisfaction (Figure 3A). Across all sessions, over 95% of responses indicated that the lessons “made sense”, and more than two-thirds of participants strongly agreed that they understood the ideas presented, enjoyed the lessons, and felt the pacing was appropriate. Although the majority expressed ease with the material, a notable 21% reported that the lessons were “tough”, suggesting that the content was appropriately challenging while still accessible.

Daily reflections reported by learners most frequently described themselves as knowledgeable, well-informed, empowered, and educated (Figure 3B). Many also reported feeling great, awesome, excited, inspired, or rejuvenated, and several highlighted gaining “great knowledge,” feeling “ready for action,” and being “skilfully prepared.” Overall, the responses reflect a strong sense of satisfaction, personal growth, and motivation among participants by the end of each training day.

### Quantitative evaluation: program evaluation survey

**Figure 4:**
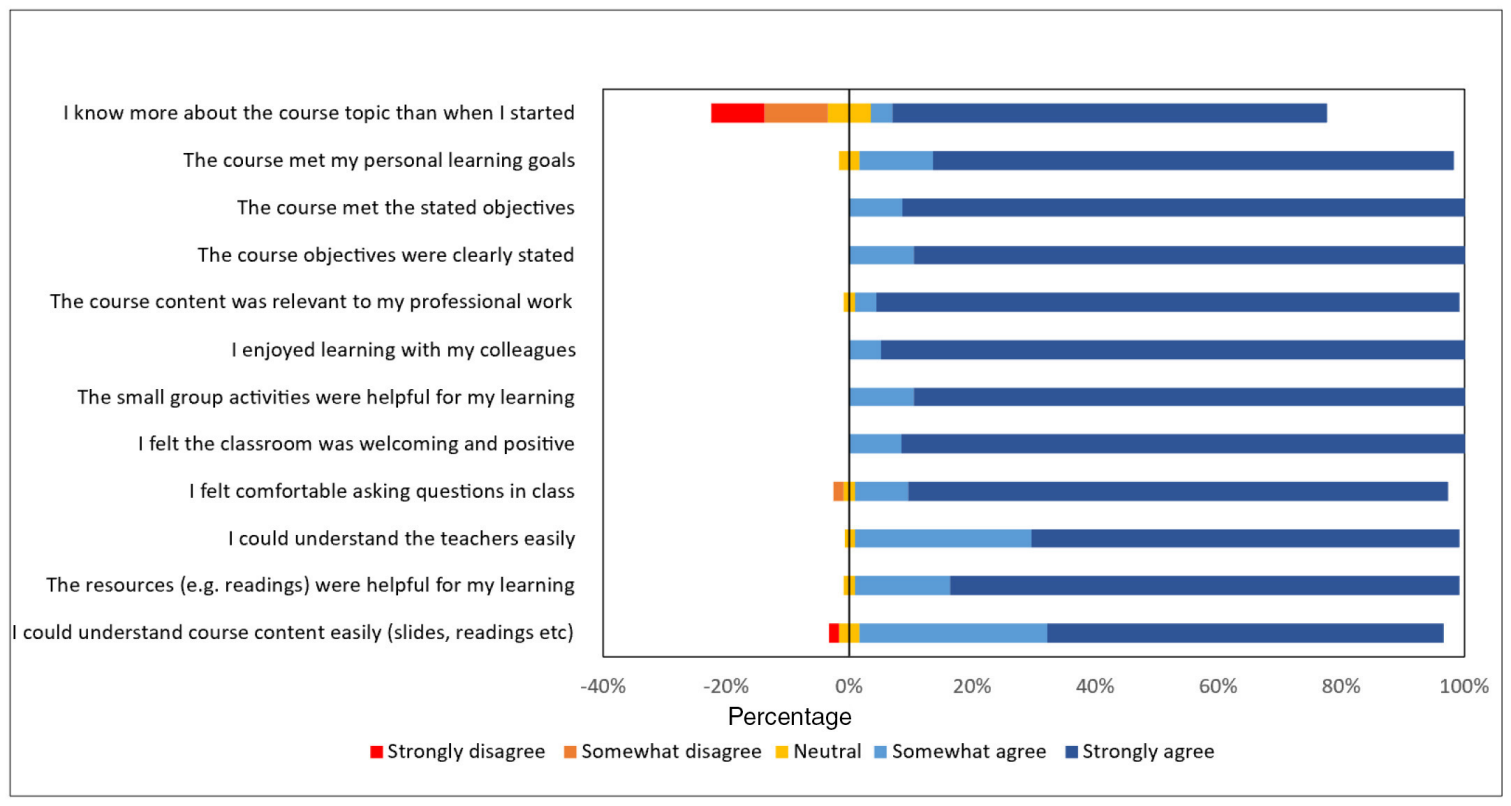
Overall evaluation, 2023–2025 (*n* = 59).

Fifty-nine of 65 participants (91%) completed the end-of-course evaluation survey. Participants reported positive experiences with the course (Figure 4), with 50 (98%) indicating they were extremely or somewhat satisfied, 59 (100%) rating the teaching as extremely well or very well, and 58 (98%) stating they were extremely or somewhat likely to recommend the course to a colleague. 56 (95%) strongly or somewhat agreed that they could understand the course content, 57 (97%) found the resources helpful, 58 (98%) reported they could understand the teachers easily, 56 (95%) felt comfortable asking questions, and 59 (100%) agreed that the classroom environment was welcoming and positive. While 48(81%) reported enjoying learning with colleagues, 56 (95%) felt the content was relevant to their professional work, 57 (97%) agreed the objectives were clearly stated, 58 (98%) agreed the course met its stated objectives, 57 (97%) reported meeting their personal learning goals, and 45(76%) indicated they knew more about the course topic at the conclusion than when they started.

Participants reported strong intentions and readiness to apply what they learned. All of the participants strongly or somewhat agreed that they felt confident applying the course content to their own work. Likewise, all participants indicated that they had a concrete plan to implement the course content. Most participants (54; 96%) reported an ability to identify potential challenges to material application, with only 3 learners expressing neutrality or disagreement. Perceived support from workplace supervisors was also high, with 53 (95%) strongly or somewhat agreeing that their supervisors supported their use of course knowledge.

### Qualitative evaluation: focus groups

#### Learner qualitative course feedback: structure, duration, and content

A total of six focus groups were conducted across the three cohorts (two per cohort), with approximately 20 participants per group. Both supportive and critical perspectives were identified across focus groups. While participants consistently described positive learning experiences, they also highlighted important limitations, including the short duration of the course, the need for more clinical exposure, and challenges related to implementation in practice, such as securing institutional support and advocating for service development.

The overall tone of feedback on the training environment was positive. Many participants appreciated the interactive and engaging nature of the course, with one noting,

“*The pacing was good, and the discussion was interactive.”*

Another participant shared that the motivation provided by the course, including the accommodation, food, and overall package, was very effective, helping to foster a positive learning experience.

A significant concern voiced by participants was the short duration of the training, which many felt was not long enough to fully absorb the material. Additionally, many participants expressed a desire for more practical exposure. While some hands-on experience was offered, it was seen as insufficient. Several others suggested that a longer course (e.g., 2 weeks) with more clinical sessions would better support their learning and practical application of the skills taught. Finally, several participants expressed the desire for follow-up support to ensure that the momentum generated by the course would continue once they returned to their facilities. These findings reflect both positive reception of the course and constructive feedback regarding areas for improvement.

#### Learner qualitative feedback: application of learning

When learners were asked to reflect on the course content and learning application, the following 5 themes emerged; the importance of communication, patient advocacy for informed decision making, the holistic approach to patient care, the involvement of families and caregivers, and the practical steps required to implement palliative care into practice.

*The Importance of Communication in Palliative Care* Communication emerged as a central theme in the course. Learners emphasized the need for clear, open dialogue with patients about their conditions, especially when discussing sensitive topics like death and end-of-life care. One participant noted,

“*This has taught me that communication is key.”*

Another emphasized the value of addressing patients' fears and anxieties:

“*The training helped me to communicate… to have difficult conversations with clients, talking about death, their fears, and their anxiety.”*

Participants reflected on how they now actively engage patients in conversations, ensuring they understand their diagnoses and treatment options. One learner shared,

“*After the training, I asked one patient, do you know what you're coming in for? And the patient said no. The doctor asked me to come here with my medication.”*

This indicated a recognition of gaps in communication in their practice prior to the course.

*Patient Advocacy and Informed Decision-Making* Many learners highlighted how the course shifted their perspective on patient advocacy, particularly around the need for transparency about a patient's condition. One participant explained,

“*We have to communicate more with our patients… After the training, I started educating patients... I realized sometimes staff or health professionals; we try to hide behind the truth.”*

The course also reinforced the significance of involving patients and families in decisions, particularly regarding advance care planning and goals of care. As another participant shared,

“*I have learned that we shouldn't assume things for our patients... we should always ask questions and get to the in-depth knowledge of what the patient is going through.”*

*A Holistic Approach to Patient Care* The course also emphasized a more comprehensive approach to care that considers not just the physical but also the psychological, social, and spiritual needs of patients. This holistic perspective was felt to be crucial for improving the overall quality of life for patients, especially those nearing the end of life. One participant added,

“*This course has really opened our eyes to a total care approach, looking at their spirituality, their psychological aspects, and their social aspects, even if they're going to die, they will go peacefully.”*

*Family and Caregiver Involvement* A key takeaway for many learners was the importance of involving families and informal caregivers in the palliative care process. Learners recognized that families play a crucial role in supporting patients and that understanding family dynamics can help deliver better care.

Implementing Palliative Care in Practice This theme generated particularly rich discussion across focus groups, reflecting its central importance to participants. Learners felt more empowered to integrate palliative care into their clinical settings, with many expressing a desire to start or expand palliative care services at their facilities. One participant shared,

“*I feel more confident and empowered to start palliative care within my small clinic.”*

Another noted that the course provided practical steps, saying,

“*The second module has really given me practical ways to help me set up a unit in my department… I now feel well equipped and competent to provide palliative care in the clinical setting.”*

Despite challenges in convincing management to support palliative care, many learners felt determined to advocate for its integration, with one participant stating,

“*I will need management support to fully implement everything I have learned here, but I'm determined to do that.”* Another echoed this, saying, “*I already have management support, so integrating the services into our existing services is going well.”*

Participants also emphasized challenges to implementation, including the need for management support, resource constraints, and the importance of local advocacy to establish or expand services.

## Discussion and acknowledgements of constraints

The aim of the manuscript was to describe the development and evaluation process for an innovative co-created short course palliative care program for Ghanaian health care professionals. Overall, participants reported high satisfaction, strong perceived learning, and readiness to apply new skills, alongside identified needs for longer training, increased clinical exposure, and ongoing mentorship. These findings are encouraging as they support the opportunity for near-term practice change and informative for scale-up design to build capacity in resource-constrained settings.

Our results fit into the global context in which palliative care is recognized by the World Health Organization (WHO) as an essential component of Universal Health Coverage (UHC), yet only a small fraction of those in need receive it (6, 30). Much of the existing literature on palliative care education is derived from high-income settings, and its applicability to resource-constrained contexts such as Ghana may be limited. Sub-Saharan- African health systems face particularly acute barriers, a limited trained workforce, uneven service distribution, under availability of essential medicines, financial and geographic barriers, and persistent misconceptions about palliative care, necessitating locally tailored capacity building strategies (18). In Ghana specifically, qualitative studies document late referrals, gaps in communication, and patient perceived needs for coordinated, person-centered care (31). Our data, and the existing literature reinforce the value of training programs that strengthen symptom expertise and serious-illness communication while expanding reach beyond major urban centers. While prior literature demonstrates that educational interventions can improve communication behaviors, teamwork, and patient-level outcomes, the present study was limited to self-reported measures of learning, confidence, and intended practice change, and did not directly assess behavioral or clinical outcomes.

### Alignment with adult learning and interprofessional education evidence

The pedagogical design used blended didactics with case-based discussion, role-play, reflective exercises, site-based clinical observation, and iterative feedback. These elements of learning are consistent with established adult learning and experiential learning theories, which emphasize relevance, active engagement, reflection, and repeated application to enhance retention and transfer to practice (32). Simulation and roleplay are associated with improved teamwork attitudes and self-reported communication confidence; integrating them before or alongside simulation can further augment learning effects (33–35). Serious-illness communication curricula show promise but remain heterogeneous; recent reviews call for clearer modalities, longitudinal coaching, and standardized behavior/patient-centered outcomes. These elements were also emphasized by our learners in the evaluations, with many requesting longer program durations, increased clinical practice, and follow-up mentorship (19).

Interprofessional participation, predominantly nurses and physician assistants in our cohorts, aligns with evidence that interprofessional education can improve role clarity, collaboration, and team communication, even as the literature urges more rigorous links to patient outcomes (20). Building interprofessional competencies is especially relevant within the African-continent contexts where task-shifting and team-based care are essential to expanding access in rural and underserved settings. While these studies report improvements in communication behaviors and teamwork, our evaluation measured self-reported confidence and perceived learning rather than objective behavioral change. Our findings should therefore be interpreted as early indicators of readiness for practice change rather than direct evidence of behavioral or patient-level impact.

### Equity-oriented recruitment and geographic reach

Our selection process explicitly prioritized rurality, early career clinicians, and women, an approach that aligns with calls to rectify geographic and gender inequities in capacity building and to situate training where need is greatest (36). Concentration of participants around the host region, with gaps in more remote areas and limited participation from physicians and pharmacists, mirrors the urban concentration of services and workforce in Ghana and across sub-Saharan Africa. Any future iterations should deliberately oversample underrepresented regions and professions to mitigate such imbalances (18).

### From training to implementation: mechanisms to sustain practice change

Learners' high confidence and concrete plans to implement new skills are promising first-order signals; however, consistent with the broader literature, such findings represent self-reported readiness rather than confirmed behavioral change thus risk overreliance on short term, self-reported outcomes (20). Critics of the Kirkpatrick model urge moving beyond “reaction/learning” measures toward behavior in the workplace and system/patient results, and to complement outcome models with program evaluation and implementation frameworks that explain “*how”* and “*why”* change occurs (20).

Given learners' request for ongoing mentorship and continued clinical support, models such as hub-and-spoke tele-mentoring [e.g., Project ECHO (37, 38)] may represent a potential approach to extending learning beyond short-course training. Project ECHO may represent a promising but context-dependent next stepto sustain skills, reduce professional isolation, and extend expertise into rural sites. This suggestion is not directly derived from the present study data but reflects a conceptual alignment between participant-identified needs and existing educational models. In Africa (37), ECHO-based palliative care networks (ECHOPACA) have improved providers' knowledge and confidence and are being prospectively evaluated for effects on patient-reported outcomes across Ghana and neighboring countries (38). Similar programs in Kenya report enhanced skills, greater collaborative care with specialists, and reduced isolation among rural clinicians, benefits that directly map to our learners expressed needs (37). However, the applicability of such models in the Ghanaian context requires careful consideration, including variability in digital infrastructure, internet connectivity, and access to technological resources, particularly in rural and underserved settings. Integration within the existing KNUST–UofT partnership could provide a feasible platform for piloting such approaches, leveraging established faculty collaboration and program infrastructure. Potential barriers to implementation include technological limitations, competing clinical demands, and the need for sustained institutional and administrative support.

### Implications for Ghana's health system and policy alignment

WHO's framework and the Global Atlas underscore that integrating palliative care across levels of care (30) and clinical settings, along with training and essential medicines policy is foundational to UHC. Ghana's emerging policy and training initiatives [e.g., the Ghana College of Nurses and Midwives' palliative nursing programs (39) and new children's palliative care capacity building efforts (40)] signal momentum that our program can complement by upskilling frontline providers and strengthening referral pathways. Qualitative work within Ghana highlights system barriers (e.g. distance, cost, awareness, referral pathways) and the importance of meaningful communication (31). Embedding modules on serious illness conversations, shared decision-making, and caregiver engagement, all core elements of our curriculum, directly address these gaps.

### Partnership approach and co-creation

This project exemplifies pedagogical solidarity, moving beyond extractive research paradigms to foster genuine spaces of co-creation (22). The curriculum was designed collaboratively, with Ghanaian faculty as equal partners rather than subjects of study, reflecting commitment to decolonizing research practices (23). This is research “with” rather than “on”, a distinction that reshapes both the process and the knowledge produced (24).

The co-creation between KNUST and UofT reflects current guidance for equitable global health partnerships, prioritizes locally identified needs, mutual benefit, shared governance, and pays attention to power asymmetries. (25) Tools such as the Equity Tool for Valuing Global Health Partnerships (26) can facilitate ongoing reflection on governance, inclusion, and impact as the collaboration grows, and help avoid pitfalls seen in short-term or extractive models. (41) Strengthening regional ownership and leadership is also consistent with broader calls to build authentic, cross-regional partnerships that support sustainable capacity rather than episodic training. (25)

### Strengths and limitations

Strengths include intentional equity-oriented recruitment, interprofessional participation, a curriculum grounded in adult learning principles, and mixed methods evaluation capturing learner experience and planned behavior change. The primary limitations are reliance on self-reported outcomes without objective behavior or patient and system level measures, potential response bias, underrepresentation of physicians and pharmacists, and geographic imbalances toward the host region. These limitations mirror the broader palliative care education literature's need for longitudinal, multisite, and patient-centered outcome studies. As such, caution is warranted in interpreting these findings as evidence of practice change or patient-level impact.

This study has several important limitations. First, the evaluation did not include a control group or pre–post assessment of knowledge or skills, limiting the ability to determine the magnitude of learning gains or attribute observed outcomes directly to the intervention. Second, findings are based largely on self-reported data, and responses, particularly within focus groups, may be subject to social desirability bias, as participants were known to facilitators and may have been influenced by group dynamics. Third, while participants expressed readiness and intention to implement palliative care practices, no longitudinal follow-up was conducted to assess actual changes in clinical practice or patient-level outcomes. Potential unintended or downstream effects, particularly in the context of constrained health workforce capacity should be considered, and future program development should consider system-level supports, resource allocation, and role integration to ensure sustainable implementation. Finally, although the program aimed to engage a broad interprofessional audience, physicians and pharmacists were not represented among participants, limiting generalizability and highlighting ongoing challenges in engaging these groups in palliative care training initiatives.

## Conclusion

In a setting with substantial unmet palliative care need and workforce constraints in Ghana, an innovative co-created, equity-oriented, interprofessional short course in Palliative Care was a welcomed first step to position participants to implement their newly skills. Anchored in a primary health care (PHC) approach, this model strengthens the capacity of frontline providers to deliver person-centered palliative care within communities. Aligning scale-up with adult learning best practices, equitable partnership principles, and implementation frameworks, offers a practical path to extend palliative care capacity across Ghana's regions while contributing to stronger PHC systems, advancing Universal Health Coverage, and generating the longitudinal, patient-centered outcomes increasingly called for in the field.

## Data Availability

The datasets presented in this article are not readily available because no datasets available. Requests to access the datasets should be directed to NA at NA@google.com.
